# In Situ Synthesis of AZO-Np in Guar Gum/PVOH Composite Fiber Mats for Potential Bactericidal Release

**DOI:** 10.3390/polym14224983

**Published:** 2022-11-17

**Authors:** Adriana Freire Lubambo, Ney Mattoso, Lucy Ono, Gisele Gomes da Luz, Bruno Gavinho, Andressa Amado Martin, Maria Rita Sierakowski, Cyro Ketzer Saul

**Affiliations:** 1 Department of Physics, LITS, Universidade Federal do Paraná-UFPR, Centro Politécnico, Curitiba P.O. Box 19044, PR, Brazil; 2 Department of Basic Pathology, Yasuyoshi Hayashi Microbiology Laboratory, Universidade Federal do Paraná-UFPR, Centro Politécnico, Curitiba P.O. Box 19031, PR, Brazil; 3 Department of Chemistry, Biopol Laboratory, Universidade Federal do Paraná-UFPR, Centro Politécnico, Curitba P.O. Box 1908, PR, Brazil

**Keywords:** AZO-Np, guar gum, nanoparticle regrowth, composite membrane electrospun

## Abstract

Since the number of antibiotic-resistant bacterial infections is growing and cases are getting worse every year, the search for new alternative bactericidal wound dressing treatments is becoming crucial. Within this context, the use of polysaccharides from plants and seeds in innovative biopolymer technologies is of key importance. In this work, bio-nano-composite guar gum/polyvinyl alcohol (PVOH) membranes loaded with aluminum-doped zinc oxide nanoparticles were produced via electrospinning. Citric acid was added to the mixture to increase spinnability. However, depending on the pH, zinc oxide nanoparticles are partially dissociated, decreasing their bactericidal efficiency. Thus, a second successful alkaline thermo-chemical regrowth step was added to the process to treat the obtained fibers. This alkaline thermo-chemical treatment reconstituted both the nanoparticles and their bactericidal properties. The *Staphylococcus aureus* antibacterial assay results show that the membranes obtained after the alkaline thermo-chemical treatment presented a 57% increase in growth inhibition.

## 1. Introduction

Recent studies show that the number of bacterial infections increases every year, and these numbers also reflect the increase in multi-drug-resistant bacterial infection deaths [1]. One of the reasons for such rising numbers is the inappropriate and persistent use of antibiotics, which leads to antibiotic resistance. It is known that bacteria can rapidly develop resistance mechanisms, such as antibiotic target site alteration, antibiotic inactivation, and metabolic changes to minimize drug entry. Therefore, the surviving bacteria adapt to thrive into the novel environment. For this reason, the search for novel antibiotics and new antibacterial materials is crucial.

Within this scenario, there is a growing interest in developing novel nano-biocomposite materials with bactericidal properties. These systems can be exploited for wound dressing and tissue engineering, [2,3] unlike the standard antibiotics used to avoid bacterial formation. Furthermore, the use of low-cost biodegradable patches with a fast bacterial product release would allow the attenuation of incipient bacterial colony formation. Moreover, it is worth considering that their action would be superficial and would naturally dissolve on the skin with time, which provides the additional advantage of not damaging deeper skin layers. Among these novel materials, composite membranes of polysaccharides blended with synthetic polymers, such as polyvinyl alcohol (PVOH), and loaded with bactericidal nanoparticles are adequate candidates to suit this emerging area. Such materials are versatile due to their low cost, biocompatibility, and biodegradability [4,5,6]. Furthermore, their composite membranes can be easily produced via electrospinning, which is a simple and low-cost technique that produces nanofibers with a high surface-to-volume area ratio [7,8]. It simply demands a high-voltage supply, a syringe pump, a polymeric composite solution, and a grounded collector. The electrospun mats consist of non-woven ultrafine fibers formed from a dried electrostatically ejected polymeric solution thread [8,9].

Zinc oxide nanoparticles composite electrospun mats have already been re-ported in to have bactericidal applications [10,11]. This bactericidal property could rest on their peroxide formation mechanism, direct nanoparticle–cell membrane wall interaction, or a combination of both mechanisms, which are believed to be toxic to bacteria [12,13,14]. In fact, studies on gram-positive and gram-negative bacteria, along with eukaryotic nematodes, have demonstrated this [15,16]. Furthermore, a recent study about Al-doped ZnO (AZO) nanoparticles showed their temporally dependent toxicity to *Pseudomonas Putida* in a biosensor. However, when single Al/Zn ions were separately tested, no toxicity was observed [17].

There are several methods by which ZnO or AZO nanoparticles may be incorporated in a polymer matrix. The most common route is the use of a precursor in the mixed solution prior to forming the electrospun fibers [18,19,20]. An alternative manner is the incorporation of nanoparticles and polymers in a mixed solution [10,21,22] before fiber formation. Electrospinning polymer and precursor together before in situ growth via a wet chemical route is also possible [23]. Finally, plasma deposition [24], which is a route to achieving AZO thin film formation, could be a potential method to use before fiber formation by electrospinning.

Guar gum (GG) is a water-soluble polysaccharide found on the endosperm of leguminous seeds such as, for example, *Cyamopsis Tetragonaloba* [25]. It is a neutral polysaccharide whose structure are formed by a backbone of (1-4) linked β-d-mannopyranose units with random branch points of α-d-galactopyranose units. On average, there is a ratio of 1.5 to 2 mannoses for each galactose unit, as well as the occurrence of some non-substituted regions. As a water-soluble polysaccharide, GG is hydrophilic, easily available in nature, non-toxic, and biodegradable [26]. Generally, due to its high molecular weight and the occurrence of intermolecular hyper-entanglements produced by inter-chain hydrogen bonding [27], GG is used as a viscosifier, flocculant, or stabilizer in the industry [28,29,30]. Its pseudo-plastic behavior even at low concentrations makes GG suitable to be widely applied in drilling and oil recovery [30,31]. It can also easily mimic the role of hemicellulose in wood, therefore having a broad application in the paper industry as a mechanical property enhancer [32]. Nonetheless, pure GG, as a consequence of its hyper-entanglements and high viscosity, only forms fibers on the nanometer range. Its association to synthetic polymers, such as PVOH, allows for overcoming this disadvantage and producing sustainable electrospun membranes [33].

PVOH in its own turn is a water-soluble synthetic polymer widely used as a carrier because of its physicochemical stability and biocompatibility [34].

Nevertheless, during electrospinning, the simple mixture of PVOH/GG still presents agglomerates which impair both Taylor cone formation and stability; thus, the polymer must be properly dissolved in the solvent, which is essential to fiber formation [8,35]. Guar gums are also known to produce brittle films with humidity issues [36]. To improve mechanical properties and provide better mixture solubility, citric acid is used as a plasticizer in our composite mixture. Citric acid is found as a natural component of fruits. It is a non-toxic, low-cost available metabolic byproduct of the human body (Krebs cycle). Therefore, it is expected to be non-toxic to the dermal cells [34,37]. Another advantage rests in the fact that its addition increases zinc oxide nanoparticle solubility to a certain extent as zinc oxide hydrolyze, forming hydroxide layers at the surface of the nanoparticle, thus becoming slightly soluble in water at room temperature [38,39]. However, successful zinc oxide incorporation depends on the citric acid ionic strength in the solution. The reason for this is that in acidic pH, zinc oxide partially dissociates in Zn^++^ depending on the citric acid concentration [40,41]. This condition is not suitable for bactericidal purposes, since it hinders the necessary peroxide formation mechanism. One way to overcome this problem is to use a low-temperature wet chemical path to reconstitute AZO nanoparticles by oxidizing zinc cations in situ with a hot alkaline aqueous solution [10,24].

In the present investigation, we aimed to successfully produce bactericidal PVOH/GG/AZO electrospun composite membranes for fast bactericidal release patches using citric acid as a plasticizer. To overcome the partial dissolution of AZO into hydroxylated Zn cations, we performed a post-chemo-thermal treatment to restore ZnO nanoparticles in the membrane surface layers. In addition to its low cost, the advantage of this procedure is that the use of zinc oxide precursors is not necessary, which would need high temperature processing to obtain the AZO nanoparticles and therefore would degrade the PVOH/GG membranes [42,43]. We also performed bactericidal assays to check the membranes’ efficiency.

## 2. Materials and Methods

### 2.1. Materials

Commercial (GG) was purchased from Biotec (batch No 21038, Rio de Janeiro, Brazil) with average molar mass (Mw: 519,000 g/mol). It was purified and characterized as in Lubambo et al. [44].

PVOH with average molar mass (Mw: 140,828 g/mol), 86.5–89.5% hydrolysis, 40–48 mPa.s, polydispersity index (Mw/Mn: 1.65), and intrinsic viscosity ([η]: 0.737 dl/g) was purchased from VETEC (Brazil) whose physic chemical characteristics were determined by a Viscotek GPC and which was coupled to a multi-detector system. The Mark–Houwink constant (α: 0.472) corresponding to a random-coiled polymer was consistent with the literature [45].

Commercial aluminum-doped zinc oxide nanoparticle was purchased from Sigma Aldrich with 6% Al as dopant; nanoparticles were smaller than 50 nm and had surface area greater than 10.8 m^2^/g according to the technical note from the manufacturer.

All other chemicals were P.A. grade and were used as purchased. NaOH and citric acid was from Vetec. Ethanol was from Biotec.

### 2.2. Solution Preparation Procedures

- Solubilization of PVOH and GG at pH 7:

A total of 1.05 g of PVOH was stirred with distilled water at 60 °C for 30 min in a 10 mL beaker. Afterwards, the hot plate was turned off and the solution continued to be stirred for 24 h.

In another 10 mL beaker, 0.42 g of GG was stirred in distilled water for 24 h at room temperature. After 24 h of GG stirring, aluminum-doped zinc oxide nanoparticles were added to the solution according to Table S1 (Supplementary Materials) and stirred for another 24 h.

After these solubilization procedures, both beaker contents were mixed in a 1:1 ratio and stirred for 30 min. The final mixture had a pH 7 determined by using a pH indicator paper from Merk.

- Solubilization of PVOH and GG at pH 7:

The solubilization of PVOH and GG followed the same protocol as for pH 7 except that 0.5 mg of sodium borohydride was added previously to the GG/AZO-Np mixture before the addition of PVOH solution to protect the glycosidic terminals against alkaline degradation (β-elimination).

When GG/AZO-Np and the borohydride were solubilized, 1 M NaOH drops (2.5 μL) were added while stirring until the mixture reached pH 10. Finally, both GG/AZO-Np/borohydride and PVOH solutions were mixed in a 1:1 ratio and stirred for 30 min, the pH was corrected until it reached 8 with drops of 0.5 M NaOH, and the whole solution was sonicated using a Branson ultrasonics™ sonifier™ SFX250 with a 1/2” diameter tapped bio horn and 1/2” extension. It was sonicated with amplitude of 30%, (10 s on/10 s off) cycles for 30 min, before electrospinning.

- Solubilization of PVOH and GG at acidic pH:

The solubilization of PVOH and GG followed the same protocol as for pH 7 except that citric acid was added to the PVOH beaker while stirring before mixing with GG solution according to Table S1 (Supplementary Materials). Finally, both beakers were mixed in a 1:1 ratio and stirred for 30 min before use.

### 2.3. Electrospinning

The positive terminal of a high-voltage power supply (0–40 kV) was attached to a rectified (blunt tip) 22-gauge standard syringe needle, which worked as a metallic capillary. The mixture solution was loaded into a 2.5 mL gastight Hamilton syringe and pumped with a homemade syringe pump at (31 ± 6) µL/min flow rate. The applied voltage was (16.0 ± 0.5) kV. The tip-to-collector distance was (20.0 ± 0.3) cm. These parameters (voltage, flow rate, and tip–collector distance) were adjusted to obtain a stable Taylor cone during the electrospinning process. We used a grounded polished aluminum collector plate. The electrospinning deposition around 45% room relative humidity was finished when the substrate collector was covered with a self-sustained membrane. The obtained membranes were manually peeled from the metallic collector.

### 2.4. Chemical and Thermal Treatments of Membranes

After deposition, all membranes were stabilized by thermal treatment [46]. They were all heated at 150 °C in a vacuum chamber for 1 h at 0.05 MPa.

The membranes originated from the acidic mixture and thermally treated were also hydrothermally cross-linked in alkaline medium in a 0.1 M NaOH/ethanol solution for 30 min at 70 °C to regrowth ZnO-type nanocrystals [47]. The temperature varied by ± 1 °C during treatment. Then, they were dried at room temperature for 24 h over a Teflon substrate.

### 2.5. Bactericidal Assay Membranes Protocol

Two acidic stock solution obtained membranes were entirely cut into 6 mm diameter disks forming two groups. Each group was equally divided and stacked up into four samples, which were then pelletized with a pressure of 8 tons for 3 min. All four samples on average weighed 0.07 g. The first group of four samples was used as obtained. The disks obtained in the second group were thermo-chemically treated to regrowth AZO nanocrystals before they were stacked up to a weight of 0.07 g and pelletized.

### 2.6. Scanning Electron Microscopy (SEM) and Chemical Analysis

Images were obtained in a JEOL 6360-LV and a Tescan VEGA3 LMU, both using 15 kV as working tension. Before the analyses in SEM the samples were covered with a thin layer of carbon to enhance the images. Cathodoluminescence (CL) images and emission spectra and EDS spectra were acquired with 15 kV working tension.

### 2.7. Transmission Electron Microscopy (TEM)

TEM images were obtained using a JEOL JEM 1200 EX-II transmission electron microscope operating at 100 kV. The images were recorded using an Orius SC1000 B Gatan CCD camera. The samples were deposited directly on 200 mesh copper grids to observe in TEM.

### 2.8. Fourier Transform Infrared (FTIR-ATR)

Spectra were obtained using an ALPHA-P Bruker spectrometer with an ATR (attenuated total reflection) platinum-diamond crystal analyzer with a resolution of 4 cm^−1^. The signal was obtained from an average of three series of 24 scans from 400 to 4000 cm^−1^ and subtracted from the background. The background was obtained from one series of 24 scans. Apodization was performed using the 3rd term of a Black–Harris function.

### 2.9. Rheology

Experiments were performed using a Haake RS 1 rheometer with a cone-and-plate geometry (60 mm diameter, 2 º cones). Temperature was controlled (25 °C) by a circulating water bath Haake (DC30). Viscosity and viscoelastic property parameters were analyzed with Haake rheometer software.

### 2.10. Antibacterial Assay

The inhibition of *Staphylococcus aureus* growth was evaluated by determining the bacterial colony-forming unit (CFU) number of a suspension put in contact with the test films in contrast to a control suspension without contact with films. The bacterial suspension was prepared according to the 0.5 McFarland scale and diluted to present a final concentration of 6 × 10^4^ UFC/mL in Mueller–Hinton Broth (MHB). Commercially available bacterial cellulose membrane (BCM), (PVOH/GG/CA) (5/2/0.7) (*w*/*w*) %, 0% AZO-Np membrane with citric acid, (PVOH/GG/CA) (5/2/0.7) (*w*/*w*) % without AZO-Np membrane with alkaline treatment, or bacterial suspension were only used as a control of growth. Then, the test films were incubated with (PVOH/GG/CA/AZO-Np) (5/2/0.7/2) (*w*/*w*) %, 2% AZO-Np membrane with citric acid, (PVOH/GG/CA/AZO-Np) (5/2/0.7/2) (*w*/*w*) %, 2% AZO-Np membrane with alkaline treatment, and the controls with the bacterial suspension at 37 °C for 2 h. Bacterial growth was quantified by plating different suspension dilutions before and after 2 h of contact with films in plate count agar (PCA). The CFU number was determined after incubation at 37 °C for 24 h [48]. This experiment was run in quadruplicate and the percentage of inhibition of the test films was defined in relation to the control without film. The results were analyzed by Student’s *t*-test with *p* < 0.01. Variables exceeding the upper quantification limit were considered statistically significant.

## 3. Results and Discussion

### 3.1. SEM and TEM Analysis

**Figure 1:**
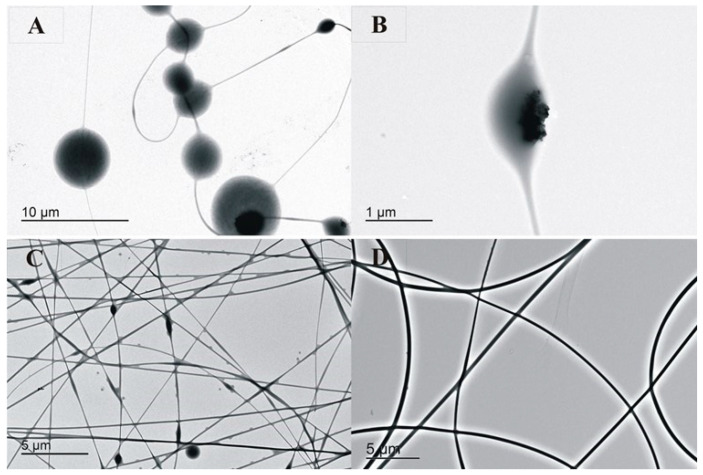
(**A**) (PVOH/GG/AZO-Np) (5/2/0.25) (*w*/*w*) %, neutral pH, 800×. (**B**) (PVOH/GG/AZO-Np) (5/2/0.25) (*w*/*w*) %, neutral pH, 5 kX. (**C**) PVOH/GG/AZO-Np) (5/2/0.25) (*w*/*w*) %, alkaline pH, 1 kX. (**D**) (PVOH/GG/AZO-Np) (5/2/0.25) (*w*/*w*) %, acidic pH, 800×.

PVOH/GG/AZO-Np membranes were produced using three different pHs, as can be seen in Figure 1. When the solution was neutral (pH 7), as shown in Figure 1A,B, it produced bead aggregates, with some of them partially filled with AZO-Np, as seen in Figure 1B. It is known that ZnO is poorly soluble in water at room temperature; it becomes more soluble if the pH is changed [39]. When the pH increased, it improved fiber homogeneity compared to the neutral condition, as seen in Figure 1C.

However, aggregates were still present over the fibers. When citric acid was added to the mixture, reducing the pH as shown in Figure 1D, a great improvement in fiber homogeneity as well as in its production rate was observed. Moreover, this result shows that citric acid helped to improve fiber homogeneity when used together with guar gum [49].

**Figure 2:**
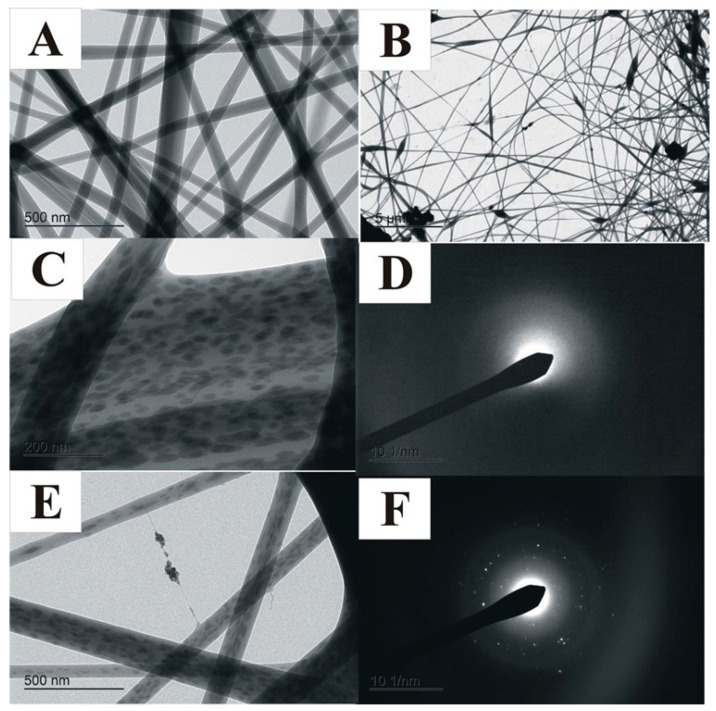
(**A**) TEM image from (PVOH/GG/CA/AZO-Np) (5/2/0.7/0.5) (*w*/*w*) % fibers mixture protocol with sonication. (**B**) TEM image from (PVOH/GG/CA/AZO-Np) (5/2/0.7/1) (*w*/*w*) % fibers with the same mixture protocol as (**A**). (**C**) TEM image from (PVOH/GG/CA/AZO-Np) (5/2/0.7/1) (*w*/*w*) % fibers without sonication. Black ellipsoidal spots are seen on fiber surface, 30 kX. (**D**) Corresponding diffraction pattern on the spot (**C**). (**E**) TEM image from (PVOH/GG/CA/AZO-Np) (5/2/0.7/1) (*w*/*w*) % fibers, nanoparticles are seen, 15 kX. (**F**) Corresponding diffraction pattern on the spot (**E**).

When AZO-Np weight concentration increases from 0.25 to 0.5 (*w*/*w*) % at constant citric acid concentration (acidic pH) and after the sonication protocol, the fiber morphology presents smoothness and homogeneity, as can be observed in Figure 2A. When the AZO-Np concentration was increased to AZO-Np 1 (*w*/*w*) %, we noticed the appearance of aggregates, as shown in Figure 2B. A further increase in citric acid concentration would restore fiber homogeneity. However, since citric acid also has bactericidal properties, [50] we decided to keep its concentration under the bactericidal threshold level so as to only evaluate the effect of AZO-NPs. It is worth pointing out that when the mixture with AZO-Np 1 (*w*/*w*) % was not sonicated, dark ellipsoidal areas on the surface of the fiber could be observed, as shown in Figure 2C. The diffraction image of the spots shown in Figure 2D shows a large halo which corresponds to semi-crystalline materials, such as PVOH and GG. Figure 2F shows a diffraction pattern that corresponds to the AZO-Np cluster, Table S2, in the center of Figure 2E. This result leads to the conclusion that the darks areas cannot be associated with the AZO-Nps, since the Nps have quite a different diffraction pattern. The dark spots are probably associated with the product of semi-crystalline ZnO lixiviation by citric acid. The darkening of the spot is related to atomic numbers greater than PVOH/GG that constitute the fiber. This leads to higher electron absorption and a consequent darkening of the image.

Figure 2E shows the changes to dark spot geometry after sonication; according to the previous described protocol, they become thinner, straight, and elongated, meaning that sonication leads to a better fiber homogeneity. These features also indicate that the AZO-Np powder is crystalline.

### 3.2. Cathodoluminescence

**Figure 3:**
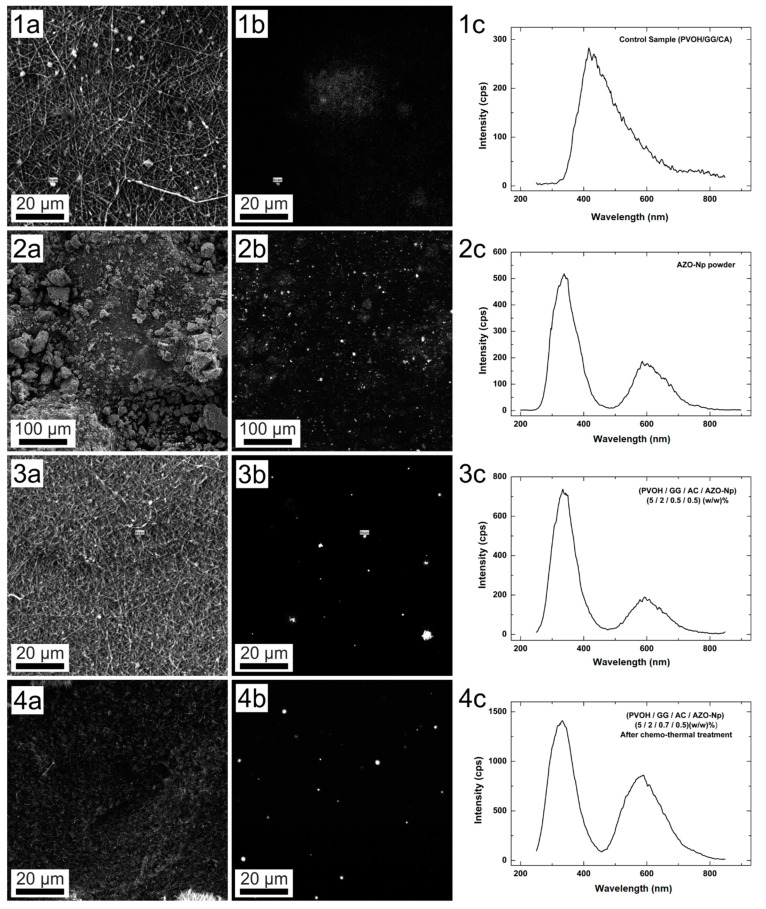
(**1a**) SEM image of membrane without AZO-Np, 1 kX. (**1b**) CL image of membrane without AZO-Np, 1 kX. (**1c**) Cl point spectrum (intensity x wavelength), membrane without AZO-Np. (**2a**) SEM image of AZO-Np powder, 200×. (**2b**) Cl spectrum of AZO-Np powder, 200×. (**2c**) Cl point spectrum (intensity × wavelength) of AZO-Np powder. (**3a**) SEM image of acidic membrane (PVOH/GG/CA/AZO-Np) (PVOH (5/2/0.7/0.5) (*w*/*w*) %, 1 kX. (**3b**) Cl spectrum of acidic membrane, 1 kX. (**3c**) Cl point spectrum (intensity × wavelength) of acidic membrane. (**4a**) SEM image of membrane (PVOH/GG/CA/AZO-Np) (5/2/0.7/0.5) (*w*/*w*) % after thermochemical treatment, 1 kX. (**4b**) Cl spectrum of membrane after thermochemical treatment, 1 kX. (**4c**) Cl point spectrum (intensity × wavelength) after thermochemical treatment, 1 kX.

Figure 3 depicts from left to right SEM images, Cathodoluminescence (CL) images, and CL spectra obtained from a selected region of different samples.

The AZO-Np powder measurement has the SEM image presented in Figure 3(2a). Its corresponding CL image shown in Figure 3(2b) presents an almost homogeneous brightness with high intensity spots distributed all over the image. Its spectrum shown in Figure 3(2c) presents two well-defined peaks, being the most intense at 339.1 nm, which corresponds to 3.66 eV band gap energy. The band gap for pure ZnO is around 3.25 to 3.28 eV. The blue shift of the nanoparticles is a clear signature of Al doping [51]. The second peak centered at 610.9 nm corresponds to defect bands, an excess of oxygen, and OH groups [52].

The control PVOH/GG/CA membrane SEM image is shown in Figure 3(1a). Its CL image is very dim as can be observed in Figure 3(1b), and its CL spectrum, shown in the graph of Figure 3(1c), presents one broad peak, which is believed to be mostly from PVOH luminescence. In fact, PVOH is the major membrane constituent and shows a visible (400–500 nm) emission due to electronic transitions in the –OH groups [53].

The untreated PVOH/GG/CA/AZO-Np membrane SEM image is shown in Figure 3(3a). Its CL image is mostly dark with large scattered bright spots, shown in Figure 3(3b). Its CL spectrum, shown in the graph of Figure 3(3c), is quite similar to the powder spectrum since the membrane has AZO-Np embedded. However, the reduced bright spot is an expected result because the AZO-Np partially dissociates in acidic environments depending on the ionic strength.

The thermo-chemically treated sample SEM image is shown in Figure 3(4a). Its CL image is shown in Figure 3(4b) and similarly to the untreated one is mostly dark with less bright spots and with more intense emission as can be observed in the CL spectrum of Figure 3(4c). Furthermore, its spectrum shows a more pronounced contribution from the defect band, around 600 nm (orange), [52] when compared with the untreated membrane spectrum, as shown in Table S2. This might be evidence that the thermochemical process reconstitutes new AZO-Nps with a higher defect concentration, as will be shown ahead with further evidence. All CL spectra were obtained with the bean focused on a single nanoparticle or with the microscope characteristic spot size. The normalized spectra are shown in Figure S1 (Supplementary Materials).

**Figure 4:**
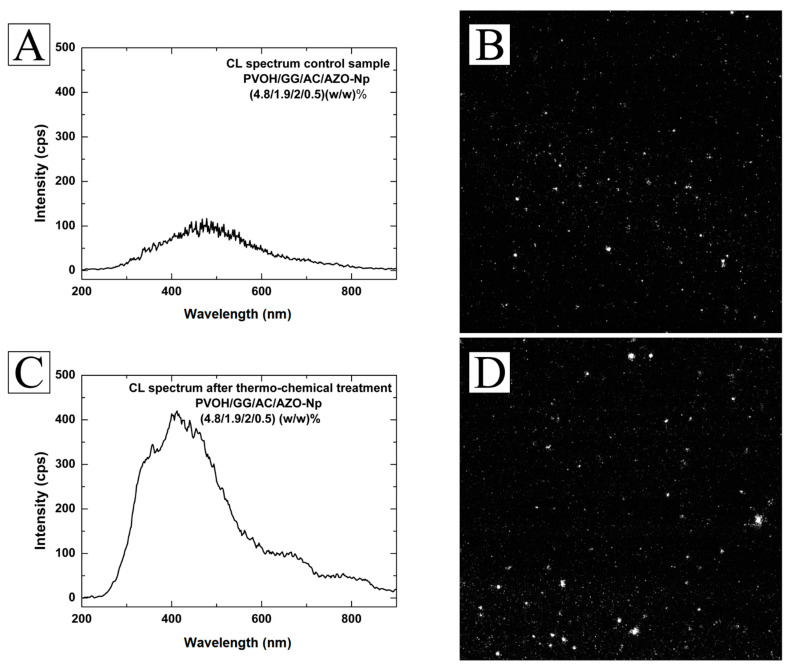
(**A**) CL spectrum obtained from a membrane with a three-fold increase in citric acid content. (**B**) CL image from the same membrane filtered at 380 nm, 200×. (**C**) CL spectrum from the thermo-chemically treated membrane showing great change due to AZO-Np regrowth. (**D**) CL image from the thermo-chemically treated membrane filtered at 380 nm, 200×.

Increasing the citric acid content from 0.7 (*w*/*w*) % to 2 (*w*/*w*) % in the mixture, that is, nearly a 3-fold increase, dissolves the AZO-Nps almost completely. The result of this dissolution is shown in Figure 4A,B for the acidic sample membrane (PVOH/GG/CA/AZO-Np) (4.8/1.9/2.0/0.5) (*w*/*w*) % where the maximum peak intensity is around 100 cps for a 200× magnification. The same sample after thermochemical treatment in a 0.1 M NaOH ethanolic solution for 1 h at 70 °C presents a completely different spectrum obtained with the same magnification, as shown in Figure 4C,D. The peak intensity around 380 nm indicates AZO-Np regrowth.

### 3.3. EDS Spectra

**Figure 5:**
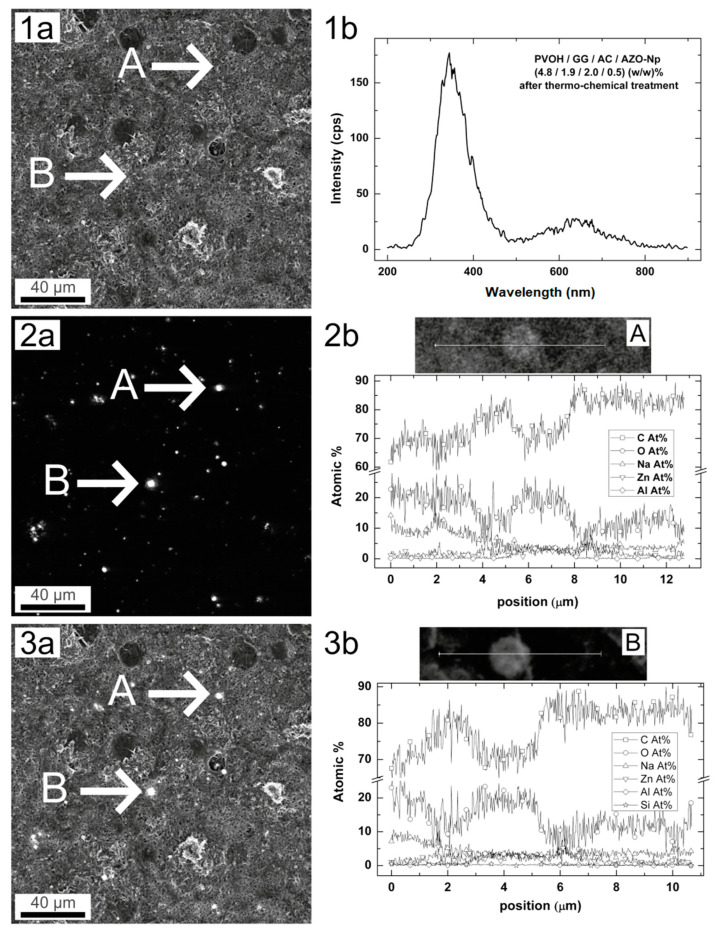
(**1a**) SEM image sample (PVOH/GG/CA/AZO-Np) (4.8/1.9/2/0.5) (*w*/*w*) % after thermo-chemical treatment, 500×. (**2a**) CL image same sample showing two corresponding luminescence points, A, B, in (**a**). (**3a**) CL image and SEM image together showing the point location. (**1b**) Cl point spectrum (intensity × wavelength) on point A, sample (PVOH/GG/CA/AZO-Np) (4.8/1.9/2/0.5) (*w*/*w*) % after thermo-chemical treatment. (**2b**) EDS elemental line profile analysis on reconstituted nanoparticles on point A. (**3b**) EDS elemental line profile analysis on reconstituted nanoparticles on point B.

The EDS spectra from the AZO-Np powder (data not shown) indicate that the original nanoparticle powder was made of 50.35 at% O, 16.53 at% Na, 2.38 at% Al, 0.15 at% Si, and 30.59 at% Zn. The presence of important concentration of sodium probably is due to chemical processes of synthesis which are not mentioned by the manufacturer. The presence of silicon at this concentration can be considered as an impurity. Figure 5 presents the SEM image of a selected region Figure 5(1a), its corresponding CL image Figure 5(2a), and the superimposition of both Figure 5(3a), as well as the CL spectrum of one of the bright spots Figure 5(1b) and the EDS profile of two selected points in samples A Figure 5(2b) and B Figure 5(3b). The sample with a three-fold increase in the citric acid content sample (PVOH/GG/CA/AZO-Np) (4.8/1.9/2.0/0.5) (*w*/*w*) % after thermo-chemical treatment was analyzed by EDS at the two selected points A and B. The EDS line profile analysis results corresponding to nanoparticles located at points A and B show that the nanoparticles had different elemental content from the original AZO-Np. The EDS content result for the nanoparticle at point A, Figure 5(2b), was 70 at% C, 19 at% O, 4 at% Na, 4 at% Al, and 4 at% Zn. The nanoparticle at point B, Figure 5(3b), had similar content when the EDS line profile was observed: 72 at% C, 18 at% O, 2.88 at% Na, 3.3 at% Al, 2.84 at% Zn, and 0.2 at% Si. The presence of silicon was probably due to impurities resulting from the thermo-chemical process. The fluorescent emission peaks also had an energy shift at point A, Figure 5(1b), thus indicating that the thermo-chemical treatment generated new AZO-type nanoparticles.

The corresponding EDS elemental map area with the two analyzed points, Figure S2 (Supplementary Materials) shows that zinc, oxygen, sodium hydroxide, and carbon are found spread in the analysis area, whereas aluminum atoms are specifically located in the nanoparticles. At first glance, zinc and oxygen are not homogenously spread on the analyzed area, and we see oxygen islands surrounded by zinc deposits. However, the detailed EDS analysis at Figure 5(2b,3b) shows the increase in oxygen where aluminum and zinc are present in the nanoparticle, indicating that is a kind of AZO-type nanoparticle.

Moreover, depending on the parameter process (time, temperature, sodium hydroxide concentration), nanoparticles with different constitutions are produced in the same process. Among them, it was possible to observe nanoparticles created without aluminum and nanoparticles without zinc. It is worth noting that the reconstituted nanoparticles are sphere-like, whereas the original zinc oxide nanoparticle was not (data not shown).

The EDS elemental map from the same sample before the thermo-chemical treatment is shown in Figure S3 (Supplementary Materials). It is possible to observe that the elemental map display is different to the one after the treatment, Figure S2 (Supplementary Materials). Elemental islands of oxygen or zinc presented in the sample after treatment are not observed. The major elements are homogeneously spread over the analysis area.

### 3.4. FTIR Spectra

**Figure 6:**
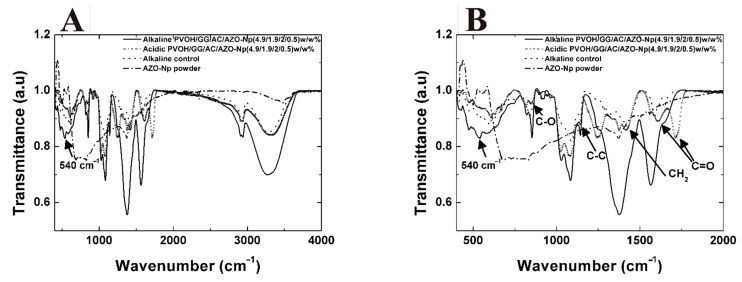
(**A**) ATR spectra of samples alkaline PVOH/GG/CA/AZO-Np (4.9/1.9/2.0/0.5) (*w*/*w*) %, acidic PVOH/GG/CA/AZO-Np (4.9/1.9/2.0/0.5) (*w*/*w*) %, alkaline control, AZO-Np nano-powder, (400–4000 cm^−1^). (**B**) Same sample transmittance spectra zoom (400–2000 cm^−1^).

Figure 6A shows the spectrum of AZO-Np nanoparticles used in these experiments.

As can be observed, these nanoparticles have absorption bands at 430 cm^−1^ and 500 cm^−1^, large bands between 526–586 cm^−1^ and between 669.7–829.8 cm^−1^, 1373.6 cm^−1^, and 1560 cm^−1^, and a broad band at 3483 cm^−1^. The spinel structure has stretching bands in the 500–900 cm^−1^ range corresponding to vibrations of metal–oxygen, aluminum–oxygen, and metal–oxygen–aluminum [54]. Peaks in the 1300–1600 cm^−1^ range are attributed to chemical impurities that come from synthesis [55], and the peaks in the 3400–3700 cm^−1^ range are attributed to chemically bonded hydroxyl vibration modes [55,56].

Figure 6B also shows the FTIR spectra of sample PVOH/GG/CA/AZO-Np(4.9/1.9/2.0/0.5) (*w*/*w*) % corresponding to the thermo-chemically treated and untreated samples and the corresponding alkaline control. The absorption peaks from the matrix PVOH/GG are described in Lubambo et al. [33]. Moreover, it is possible to observe absorption peaks for the composite fibers at 850, 1142, and 1420 cm^−1^ corresponding to functional groups C-O, C-C, and CH2 common to PVOH/GG/CA and 1611/1714 cm^−1^ C=O common to GG/CA.

As mentioned before, this sample had its citric acid content increased to dissolve the AZO-Np almost completely as shown in Figure 4A. Observing the corresponding sample spectrum, the absorption peak related to Zn-O stretching is not present in this untreated sample.

However, the corresponding FTIR spectra of the treated sample compared to the untreated sample show the appearance of one absorption peak at 540 cm^−1^ related to the new AZO-Np nanoparticles reconstituted by the thermo-chemical treatment.

### 3.5. Rheology

**Figure 7:**
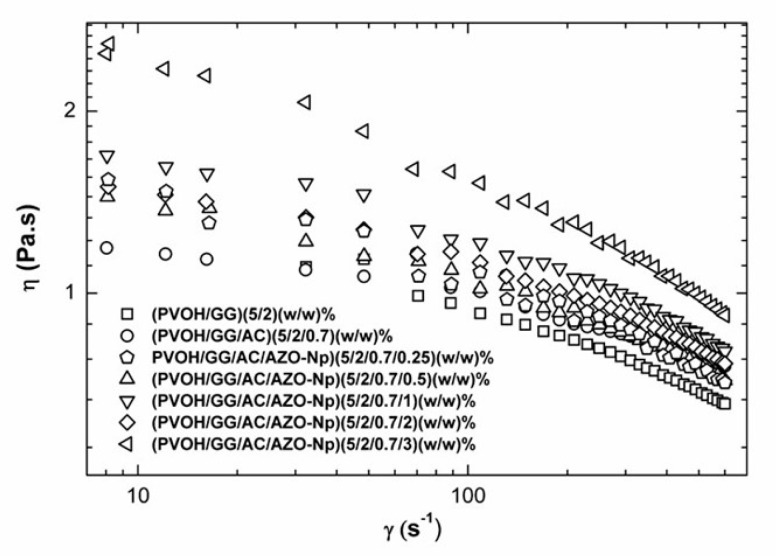
Flow curve (viscosity (η) x shear rate ($\dot{\gamma }$)) for the samples with AZO-Np in different concentrations and the controls PVOH/GG and PVOH/GG/CA.

Figure 7 below shows the flow curve (viscosity (η) x shear rate ($\dot{\gamma }$)) for the samples with AZO-Np in different concentrations and the controls PVOH/GG and PVOH/GG/CA. All samples have a pseudo-plastic behavior. It is possible to observe an increase in viscosity when the polyelectrolyte is added to the mixture PVOH/GG with or without CA, except when the AZO-Np concentration in the mixture is 5 (*w*/*w*) %. The mixture with 1 and 2 (*w*/*w*) % AZO-Np does not produce significant differences. However, increasing AZO-Np concentration to 3 (*w*/*w*) % produces a significant rise in viscosity, possibly because in this situation the interaction polymer chain water is preferential compared to polymer-AZO-Np.

The flow curves were modeled according to an Ostwald–de Waele mathematical model, which was applied to non-Newtonian fluids under shear rate. When the exponent n from the model is situated between 0 < n <1 range, they are pseudo-plastic. When n = 1, it is Newtonian [57]. Our results varied from 0.8 to 0.93 according to Table S3 (Supplementary Materials), which shows that the mixtures had a behavior very similar to a Newtonian fluid.

Since the K values as shown in Table S3 (Supplementary Materials) are proportional to the viscosity, it becomes clear that the most viscous is the sample with 3 (*w*/*w*) % AZO-Np, which is in accordance with the results in Figure 7.

The scan results of the oscillatory mode analysis as presented in Figure S4 (Supplementary Materials) display that for all the frequency rates scanned, the elastic modulus (G’) was always smaller than the viscous (G’’) one. This result is characteristic of viscoelastic dispersions with liquid behavior and is present in all the samples.

### 3.6. Antibacterial Assay

**Figure 8:**
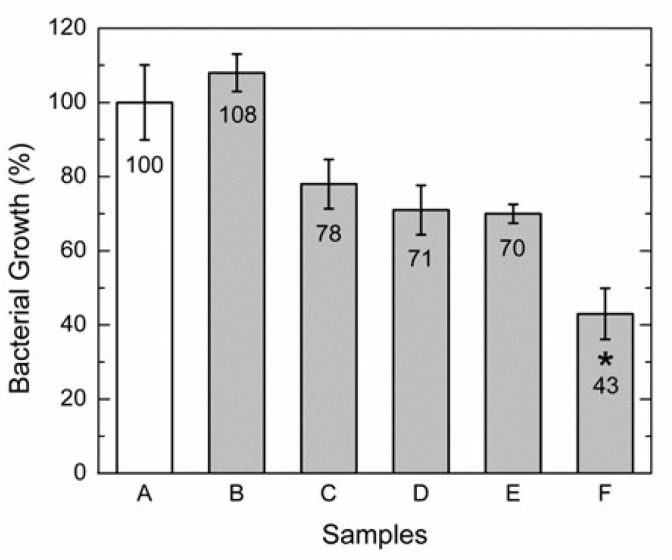
*Staphylococcus aureus* growth inhibition evaluation after 2 h at 37 °C. (**A**) Control without membrane. (**B**) Commercially available bacterial cellulose membrane. (**C**) (PVOH/GG/CA) (5/2 0.7) (*w*/*w*) % membrane with citric acid; 0% AZO-Np. (**D**) (PVOH/GG/CA) (5/2/0.7) (*w*/*w*) % membrane (the same sample as in (**C**)) with thermo-chemical alkaline treatment; 0% AZO-Np. (**E**) (PVOH/GG/CA/AZO-Np) (5/2/0.7/2) (*w*/*w*) % membrane with citric acid; 2% AZO-Np. (**F**) (PVOH/GG/CA/AZO-Np) (5/2/0.7/2) (*w*/*w*) % membrane (the same sample as in (**E**)); after alkaline treatment, 2% AZO-Np. Bars represent means, with vertical lines indicating standard deviations, * statistically significant difference when compared to control sample, n = 4, *p* ≤ 0.01.

The proper growth of *Staphylococcus aureus* occurred in the experiment controls: commercially available bacterial cellulose membrane (BCM); (PVOH/GG/CA) (5/2/0.7) (*w*/*w*) %, 0% AZO-Np membrane with citric acid; and (PVOH/GG/CA) (5/2/0.7) (*w*/*w*) %, 0% AZO-Np membrane with a thermo-chemical alkaline treatment. Therefore, no control film inhibited bacterial growth, presenting no statistically significant difference in relation to the control inoculated with bacteria without any membrane as shown in Figure 8A–D.

The sample with (PVOH/GG/CA/AZO-Np) (5/2/0.7/2) (*w*/*w*) %, 2% AZO-Np membrane with a thermo-chemical alkaline treatment, as shown in Figure 8F, inhibited about 50% of growth relative to the control without the membrane. However, the sample with (PVOH/GG/CA/AZO-Np) (5/2/0.7/2) (*w*/*w*) %, 2% AZO-Np membrane with citric acid, as shown in Figure 8E, had no activity.

## 4. Conclusions

The SEM and TEM images of the membranes with AZO-NP nanoparticles produced in neutral and alkaline pH presented bead aggregates. By adding citric acid to the mixture, the obtained fibers became more homogenous, which was probably due to the partial AZO-Np dissolution confirmed by the Zn-O stretching (540 cm^−1^) absorption band absence in the FTIR-ATR spectrum. A thermo-chemical treatment was used to reconstitute the AZO-Np nanoparticles into the electrospun fibers, and this was confirmed by the Zn-O stretching absorption band reappearance. Regarding their rheological behavior, all the mixtures were pseudo-plastic. In general, their viscosity increased with the polyelectrolyte increase into the mixture except at AZO-Np 5 (*w*/*w*) %, which is probably because there is a preferential polymer–polyelectrolyte interaction at this polyelectrolyte concentration.

The Cl spectra of the thermo-chemically treated and untreated membranes showed that they had similar spectra when compared to the AZO-Np powder. However, the treated membrane showed in its spectrum a more pronounced contribution from the defect band of around 600 nm (orange) when compared to the untreated membrane. This is a consequence of the thermochemical process reconstituting new AZO-Np with a higher number of defects. The EDS results from the reconstituted nanoparticles showed that they had different atomic percentages when compared to the original nanoparticles. It is also possible to achieve different nanoparticle content produced with the same process; the content depends on (time, temperature, sodium hydroxide content). Concerning their morphology, the reconstituted nanoparticles were sphere-like, whereas the original ones were not.

The antibacterial assays showed that *Staphylococcus Aureus* growth inhibition after 2 h at 37 °C was 30% on the membrane with citric acid when compared to the control membrane with no statistically different significance in relation to the control. However, growth inhibition increased to 57% for thermo-chemically treated membranes, indicating that the reconstituted nanoparticles increased antibacterial efficiency and therefore confirming that our membranes have a potential application as fast-release antibacterial wound dressing patches.

## Supplementary Materials

The following supporting information can be downloaded at https://www.mdpi.com/article/10.3390/polym14224983/s1. Figure S1: Normalized Cathodoluminescence from AZO-Np powder, Control membrane (PVOH/GG/CA), Membrane before thermo-chemical treatment (PVOH/GG/CA/AZO-Np) and membrane after thermo-chemical treatment (PVOH/GG/CA/AZO-Np). Figure S2: SEM image of sample (PVOH/GG/CA/AZO-Np) (4.8/1.9/2/0.5) (*w*/*w*) % thermo-chemically treated and EDS elemental map. Figure S3: SEM image of sample (PVOH/GG/CA/AZO-Np) (4.8/1.9/2/0.5) (*w*/*w*) % before thermo-chemical treatment and EDS elemental map. Figure S4: Tan (δ) values as a function of angular frequency for samples with AZO-Np in different concentrations and the controls PVA/GG and PVA/GG/CA. Table S1: Samples accordingly to their percentage weight (*w*/*w*) %. Table S2: Measured interplanar spacing dhkl from Figure 2F and known dhkl from literature. Table S3: Rheological parameters corresponding to the Oswald de Waele mathematical model.
