# Supplementary material for: In Situ Synthesis of AZO-Np in Guar Gum/PVOH Composite Fiber Mats for Potential Bactericidal Release
Source: Polymers (Basel). 2022 Nov 17;14(22):4983. doi: 10.3390/polym14224983 (PMC9698413; doi:10.3390/polym14224983)
Supplement: Supplementary file 1 [file polymers-14-04983-s001.zip › polymers-1947819-supplementary.pdf]

polymers

MDPI

In Situ Synthesis of AZO-Np in Guar Gum/PVOH Composite Fiber Mats for Potential Bactericidal Release

Adriana Freire Lubambo, Ney Mattoso, Lucy Ono, Gisele Gomes da Luz, Bruno Gavinho, Andressa Amado Martin, Maria Rita Sierakowski and Cyro Ketzer Saul

Table S1. Samples accordingly to their percentage weight (w/w)%.

Sample	PVOH (g)	GG (g)	CA (g)	AZO-Np (g)	Percentage weight (w/w)%
**Control**	1.05	0.42	0.15	-	(5/2/0.7)
**1**	1.05	0.42	0.15	0.054	(5/2/0.7/0.25)
**2**	1.05	0.42	0.15	0.108	(5/2/0.7/0.5)
**3**	1.05	0.42	0.15	0.216	(5 /2/0.7/1)
**4**	1.05	0.42	0.15	0.432	(5/2/ 0.7/2)
**5**	1.10	0.46	0.15	0.670	(5/2/0.6/3)

Table S2. Measured interplanar spacing d_{hkl} from Figure 2F and known d_{hkl} from literature*.

Measured d_{hkl} (Å)	d_{hkl} from literature (Å)
2.81±0.02	2.8143 ZnO (100)
2.58±0.02	2.6033 ZnO (002)
1.92±0.02	1.9111 ZnO (102)
1.62±0.02	1.6247 ZnO (110)

*PDF number: 000-36-1451(Crystallographica Search-Match Program).

Table S3. Rheological parameters corresponding to the Oswald de Waele mathematical model.

Sample	*n*	*K* (Pa.s^n)	r^2
PVOH/GG	0.88	1.49	0.95
PVOH/GG/CA	0.93	1.49	0.95
PVOH/GG/CA/ 0.25 % AZO-Np	0.81	2.40	0.97
PVOH/GG/CA/ 0.5 % AZO-Np	0.82	2.30	0.98
PVOH/GG/CA/ 1 % AZO-Np	0.90	1.74	0.98
PVOH/GG/CA/ 2 % AZO-Np	0.91	1.47	0.97
PVOH/GG/CA/ 3 % AZO-Np	0.80	3.71	0.99

Oswald de Waele equation: $\tau = K \gamma^{n}$.

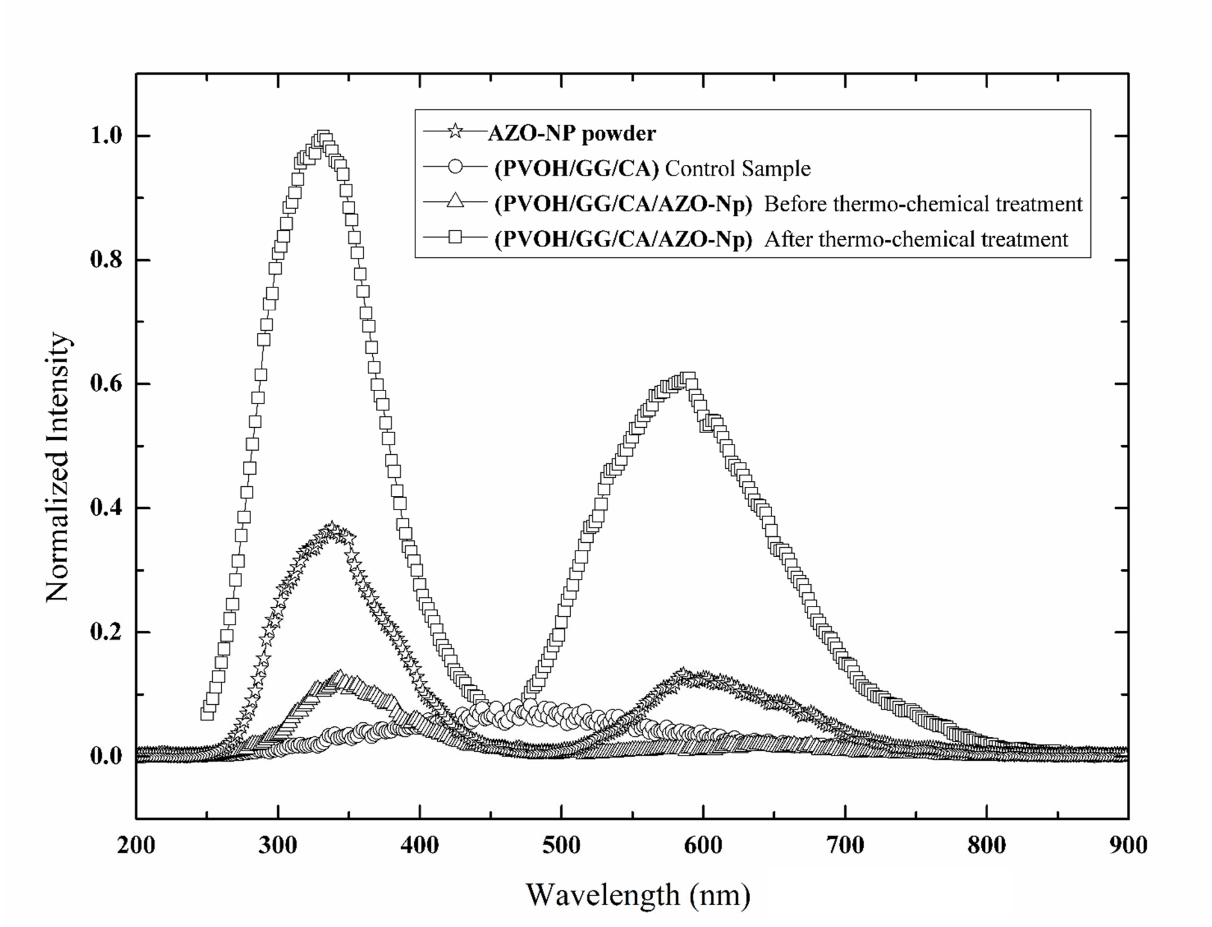

Figure S1. Normalized Cathodoluminescence from **AZO-Np** powder, Control membrane **(PVOH/GG/CA**), Membrane before thermo-chemical treatment **(PVOH/GG/CA/ AZO-Np)** and membrane after thermo-chemical treatment **(PVOH/GG/CA/ AZO-Np)**.

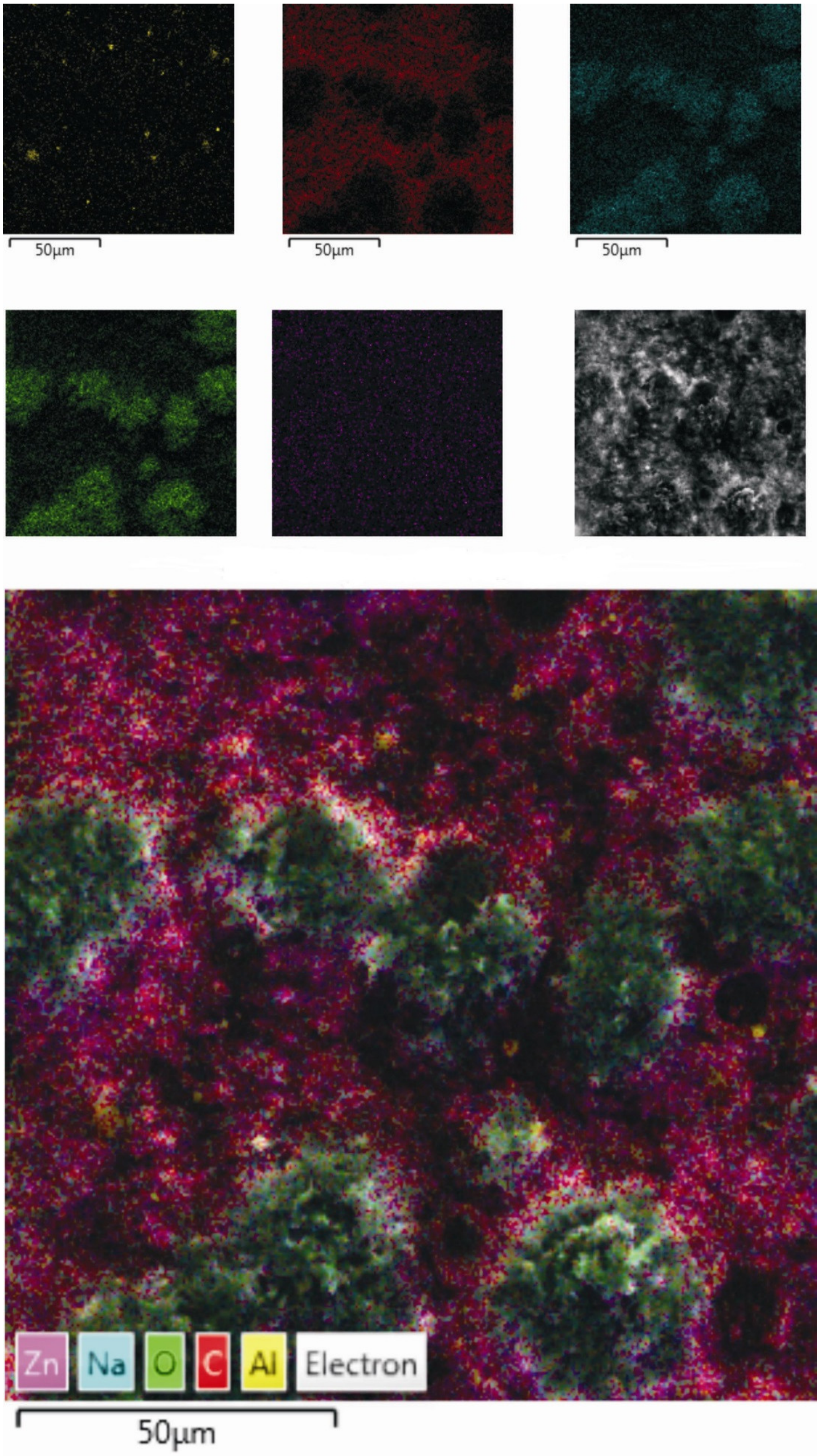

Figure S2. SEM image of sample (PVOH/GG/CA/AZO-Np) (4.8/1.9/2/0.5) (w/w) % thermo-chemically treated and EDS elemental map.

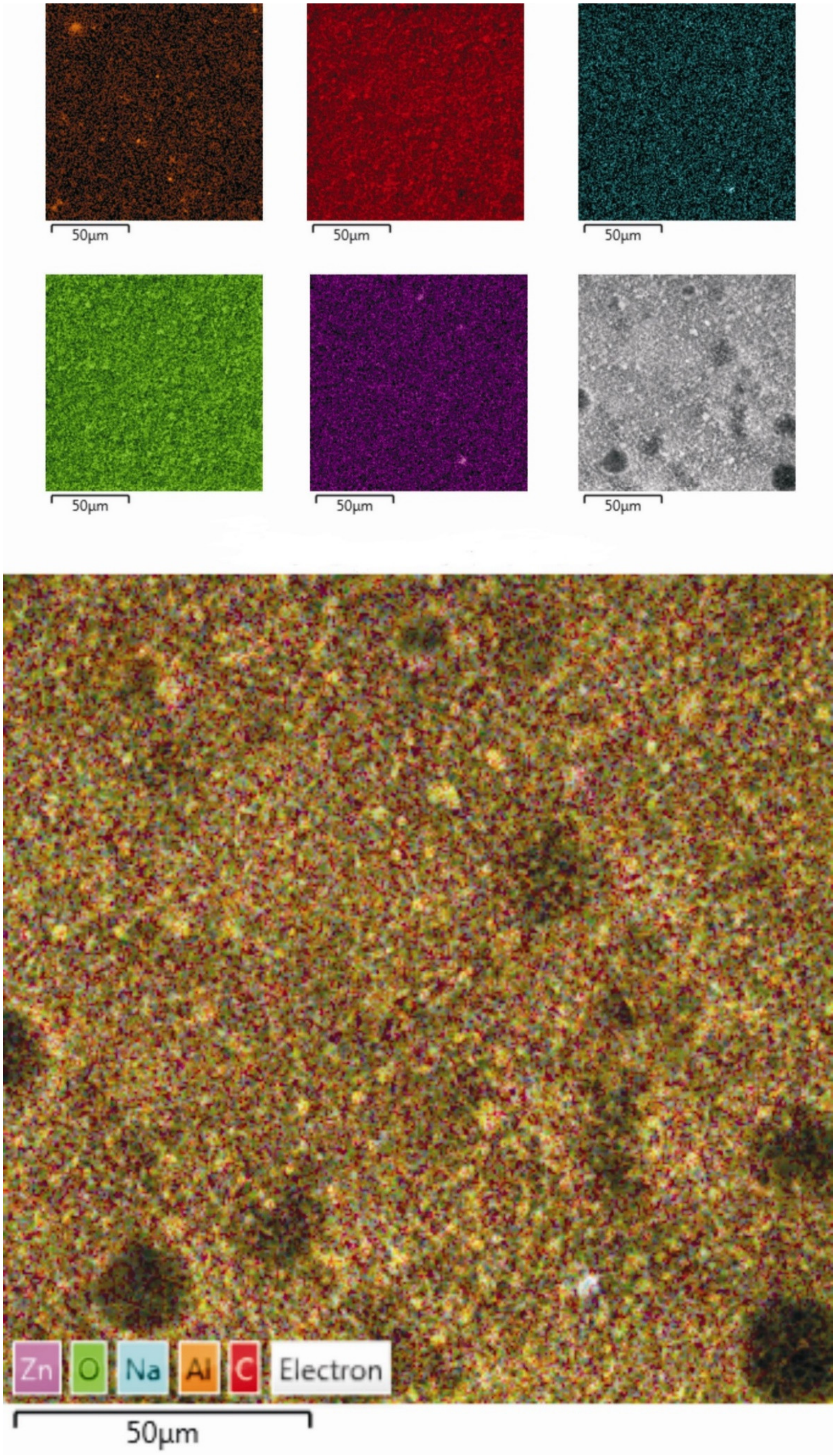
50μm
50μm
50μm
50μm
50μm
50μm
Zn
O
Na
Al
C
Electron
50μm

Figure S3. SEM image of sample (PVOH/GG/CA/AZO-Np) (4.8/1.9/2/0.5) (w/w) % before thermo-chemical treatment and EDS elemental map.

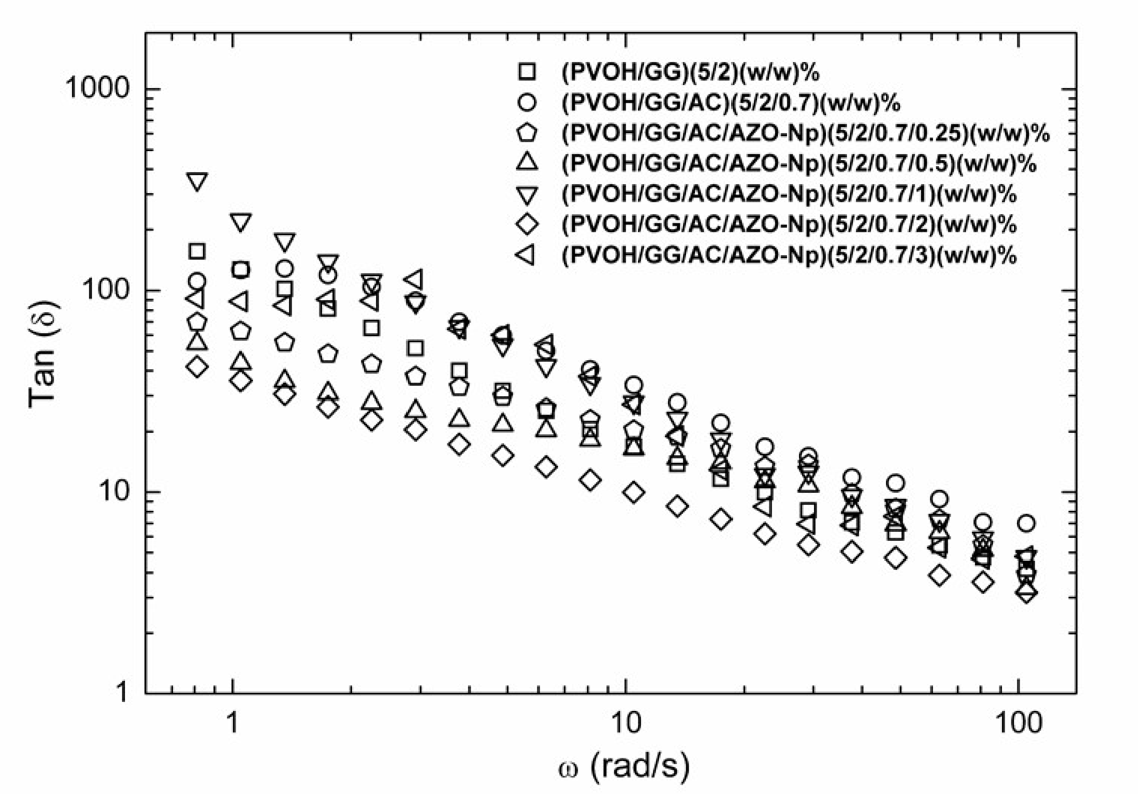

Figure S4. Tan (δ) values as a function of angular frequency for samples with AZO-Np in different concentrations and the controls PVA/GG and PVA/GG/CA.
